# Relationship between Atherogenic Indices and Carotid Intima-Media Thickness in Prediabetes: A Cross-Sectional Study from Central India

**DOI:** 10.3390/medsci6030055

**Published:** 2018-07-05

**Authors:** Roshan Kumar Mahat, Neelima Singh, Vedika Rathore, Akshara Gupta, Rakesh Kumar Shah

**Affiliations:** 1 Department of Biochemistry, Gajra Raja Medical College, Gwalior 474009, India; ved_sin26@rediffmail.com (V.R.); rakeshshah7788@gmail.com (R.K.S.); 2 Department of Radiodiagnosis, Gajra Raja Medical College, Gwalior 474009, India; aksharagupta007@gmail.com

**Keywords:** prediabetes, atherogenic indices, carotid intima-media thickness, atherogenic index of plasma

## Abstract

Prediabetes is the precursor stage of diabetes mellitus and is also considered to be a risk factor for the development of cardiovascular disease. Atherogenic indices have been used for assessment of risk for cardiovascular disease development. To date, there is no data on evaluating the relationship between atherogenic indices (cardiac risk ratio (CRR), atherogenic coefficient (AC), and atherogenic index of plasma (AIP)) and carotid intima-media thickness (CIMT) in prediabetes. Hence, we aimed to determine atherogenic indices (CRR, AC, and AIP) and CIMT in prediabetic subjects and then sought to evaluate the relationship between them. A total of 400 human subjects were included in the present study, out of which 200 were prediabetic subjects and 200 were normal healthy control subjects. For each subject, CRR, AC, and AIP were calculated from routine lipid parameters and carotid intima-media thickness was measured as well. Atherogenic indices, that is, CRR, AC, and AIP, were significantly increased in prediabetic subjects as compared to the controls (5.87 ± 0.87 vs. 4.23 ± 0.50, *p* < 0.001; 4.87 ± 0.87 vs. 3.23 ± 0.50, *p* < 0.001; and 0.29 ± 0.07 vs. 0.09 ± 0.09, *p* < 0.001, respectively). Moreover, a significant and positive correlation was observed between CIMT and AIP (*r* = 0.529, *p* < 0.01), CRR (*r* = 0.495, *p* < 0.01), and AC (*r* = 0.495, *p* < 0.01). Prediabetic subjects present abnormalities in atherogenic indices and CIMT, which indicate a greater propensity of prediabetes for the development of cardiovascular disease. Hence, atherogenic indices can be used in addition to routine lipid parameters for the better assessment of subclinical atherosclerosis in prediabetic subjects.

## 1. Introduction

Prevalence of diabetes, which is associated with an increase in morbidity and mortality, is increasing and it is one of the major healthcare problems in the world [1]. According to the pathogenesis and natural history of diabetes, it has a prolonged prediabetic phase, which is considered to be a high-risk state for diabetes [2] and can be defined by glycemic levels that are higher than normal but below the diagnostic parameters required for a diagnosis of diabetes [3]. The prevalence of prediabetes is increasing globally. In the year 2015, the International Diabetes Federation estimated that the worldwide prevalence of prediabetes was 318 million and is predicted to reach 482 million by the year 2040 [4]. In India, the prevalence of prediabetes was reported to be 10.3% in the year 2017 [5]. Prediabetes is not solely a significant risk factor for the progression of type 2 diabetes but is also considered to be a risk factor for cardiovascular disease and macrovascular disease development [6].

There is convincing evidence that prediabetes is associated with a proatherogenic lipid profile characterized by high total cholesterol, high triacylglycerol, high low-density lipoprotein (LDL) cholesterol, and low high-density lipoprotein (HDL) cholesterol, which may contribute to the progression of atherosclerosis and increase the likelihood of developing cardiovascular disease (CVD) [7,8]. Several efforts have been made in seeking emergent or new cardiovascular risk factors to improve cardiovascular disease prediction, and in an attempt to optimize the predictive capacity of the lipid profile, several atherogenic indices have been characterized. These indices can provide information on risk factors that are difficult to quantify by routine analyses and could be a more robust mirror of the metabolic as well as clinical interactions between lipid fractions [9]. One of them is the cardiac risk ratio (CRR), which is given by the total cholesterol (TC) to HDL cholesterol ratio and is frequently used for risk assessment of CVD [10]. Another index is the atherogenic coefficient (AC), which is calculated as the ratio of non-HDL cholesterol to HDL cholesterol. Non-HDL-cholesterol can be calculated easily, which does not require previous fasting of the patient. It is basically the cholesterol analogue to an apolipoprotein B level, having a better correlation coefficient in comparison with the LDL cholesterol concentration [11]. Recently, one more index, that is, the atherogenic index of plasma (AIP), was introduced, which is obtained using the logarithmic transformation of the curves that are created by dividing plasma triglyceride (TG) levels by the HDL-C levels. The AIP can be used as a better marker for atherosclerosis and assessment of risk for cardiovascular disease development [12].

Arterial wall lesions and endothelial dysfunction initiate the process of atherosclerosis, which causes an increase in intima-media thickness (IMT) [13]. This is seen in both the coronary vascular bed and the peripheral arteries [14]. Endothelial dysfunction and the increase in IMT can be determined by measurements of the carotid intima-media thickness (CIMT) [15]. CIMT is typically used to assess the cardiovascular risk of an individual, as the atherosclerotic plaques commonly develop in a region close to the common carotid artery bifurcation. Moreover, it is easily accessible with ultrasound. It has been shown that CIMT is an independent predictor of cardiovascular risk and, further, the presence of the plaques in the carotid artery is a strong predictor of cardiovascular events and mortality [16].

To date, there is no data on evaluating the relationship between atherogenic indices (CRR, AC, and AIP) and CIMT in prediabetes. Hence, we aimed to determine atherogenic indices (CRR, AC, and AIP) and CIMT, which is a reliable and early marker of atherosclerosis, in prediabetic subjects and then sought to evaluate the relationship between atherogenic indices and CIMT.

## 2. Materials and Methods

### 2.1. Study Design and Inclusion Criteria

This cross-sectional study was conducted in the Department of Biochemistry, Gajra Raja Medical College, Gwalior, India. A total of 400 human subjects were included in the present study, out of which 200 were prediabetic subjects, aged between 20–55 years old of either sex, and 200 were age- and gender-matched control subjects. The participants were selected from the general population, and those who were at risk of developing diabetes (who had at least one of the main risk factors for diabetes: first degree relative with diabetes, body mass index (BMI) ≥ 25 kg/m^2^, women who were diagnosed with gestational diabetes mellitus, women with polycystic ovary syndrome, persons who are physically inactive, and other clinical conditions associated with insulin resistance, for example, severe obesity, acanthosis nigricans, etc.) during the period of March 2017 to May 2018 in Gwalior City were identified through a predesigned screening questionnaire. The study was approved by the Ethics Committee of Gajra Raja Medical College, Gwalior, India (Ref. No: 286/Bio/MC/Ethical, Approval date: 3 March 2017), and all participants gave written and verbal informed consent consistent with the Helsinki declaration.

The prediabetic subjects were diagnosed on the basis of American Diabetes Association (ADA) guidelines as:

(a) Fasting plasma glucose level 100–125 mg/dL (Impaired fasting glucose, IFG), and
(b) 2-h plasma glucose (after giving 75 g of glucose) level 140–199 mg/dL (Impaired glucose tolerance, IGT) [17].

Those with a normal range of blood glucose level had been selected as the control group (Fasting plasma glucose (FPG) < 100 mg/dL, 2-h plasma glucose concentration after giving 75 g of glucose < 140 mg/dL).

### 2.2. Exclusion Criteria

Subjects with type 2 diabetes mellitus, cardiovascular disease, renal disease, hepatic disease, pulmonary tuberculosis, acute or chronic inflammatory disease, gout and arthritis, prolonged illness, subjects not willing to give consent or refusing to participate in the study, and patients receiving medicines known to alter glucose and lipid metabolism were excluded from the present study. Patients with triglyceride levels of 400 mg/dL and above were also excluded from the study.

### 2.3. Anthropometric Measurements

Both weight and height were measured in light clothes and without shoes using the standard apparatus. The weight was measured using calibrated electronic weighing scales prior to eating in the morning and height was measured to the nearest centimeter using a portable stadiometer. Body mass index (BMI) of the participants was calculated using standard formula; BMI = Weight (kg)/(Height (m))^2^. Waist circumference (WC) was measured using an anthropometric tape at a level on the skin midway between the mean point of iliac peak and the inferior border of the last rib at the level of the umbilicus while in a standing position at the end of gentle expiration. Hip circumference (HC) was measured over the widest part of the gluteal region at the level of pubic tubercle in standing position. Waist-to-hip ratio (WHR) was obtained by waist circumference (cm) divided by hip circumference (cm).

### 2.4. Blood Pressure Measurements

The systolic and diastolic blood pressures were taken after 10 min of resting by using a standardized mercury sphygmomanometer using standard recommended procedures.

### 2.5. Measurement of Carotid Intima Media Tthickness

The measurement of CIMT was done by using a high-resolution B mode ultrasonography system (Hitachi Aloka Medical Ltd., Tokyo, Japan) having an electrical linear transducer mid-frequency of 7.5 MHz. The patients were examined in the supine position with the neck extended and the probe in the anterolateral position. The measurements of carotid intima-media thickness were made in accordance with a previous study [18]. All CIMT measurements were performed in both the right and left carotid arteries and then the average of the right and left CIMT values were obtained. All measurements were performed by a well-trained sonographer who was blinded to all clinical data of the patients.

### 2.6. Biochemical Measurements

Five milliliters of venous blood samples were obtained from all participants under all aseptic precautions after at least 10–12 h of overnight fasting and dispensed into two different tubes based on analysis to be done. About 2 mL blood sample was taken into a fluoride bulb for estimation of fasting plasma glucose and the remaining 3 mL blood sample was dispensed into a plain bulb for analysis of lipid parameters. After that, 75 g of glucose was given orally to each participant and plasma glucose concentrations were measured at 120 min during an oral glucose tolerance test (OGTT). The collected blood samples were centrifuged at 3000 rpm for 10 min in order to get serum/plasma and all the analyses were done on the fresh serum/plasma on the same day of blood collection. All the biochemical parameters, that is glucose, total cholesterol, triglyceride, and HDL were analyzed on Mindray BS-400 chemistry analyzer (Mindray Medical International Ltd., Shenzhen, China) using commercially available kits from ERBA Diagnostics, Mannheim, Germany. LDL-cholesterol and very low-density lipoprotein (VLDL)-cholesterol were calculated using the Friedewald equation [19]. Cardiac Risk Ratio (CRR) was calculated as TC/HDL, atherogenic coefficient (AC) was calculated as non-HDL/HDL (where non-HDL is the TC-HDL), and atherogenic index of plasma (AIP) was calculated as logTG/HDL, where the concentration of TG and HDL are in mmol/L.

### 2.7. Statistical Analysis

All the data were presented as mean ± standard deviation. Statistical Package for Social Science version 20 (IBM, SPSS Statistics 20, Armonk, NY, USA) was used for data analysis. The Shapiro-Wilk test was used to check the normal distribution of data. Student’s independent sample *T*-test was used for intergroup comparisons of normally distributed parameters, whereas the Mann-Whitney U test was used for the intergroup comparisons of skewed data. A chi-squared test was used for categorical data. Pearson’s correlation was used to find the possible relationship between studied parameters. A *p* value of less than 0.05 was considered to be statistically significant.

## 3. Results

**Figure 1:**
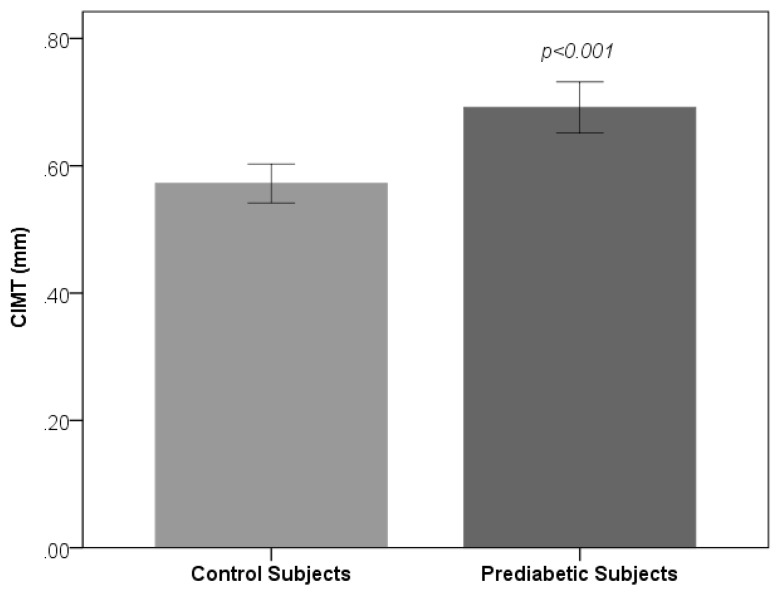
Comparison of Carotid intima-media thickness (CIMT) between control and prediabetic subjects.

**Figure 2:**
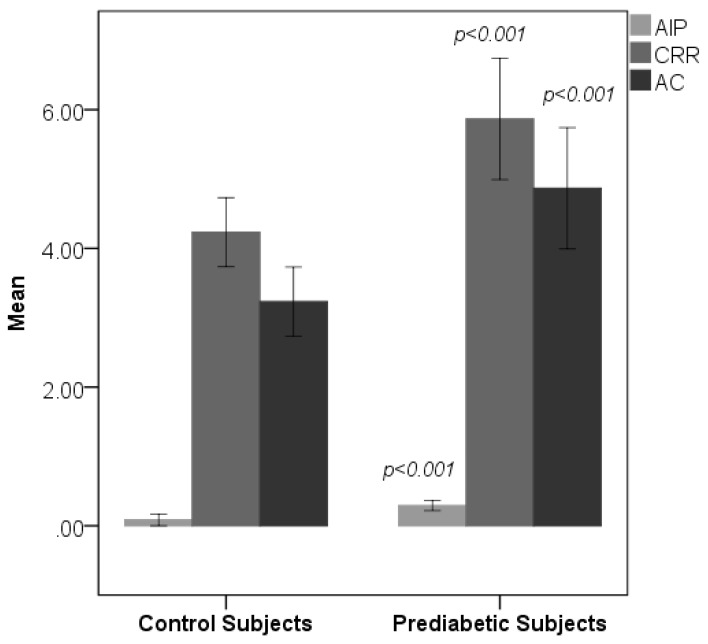
Comparison of atherogenic indices between control and prediabetic subjects. Atherogenic index of plasma (AIP) cardiac risk ratio (CRR) and atherogenic coefficient (AC).

Table 1 shows the sociodemographic and biochemical characteristics of the studied subjects. There was no difference in terms of age and gender between prediabetic and control subjects, indicating that subjects with both the groups were age and gender matched. Subjects with prediabetes had a significantly increased mean BMI, WC, HC, and WHR compared with the control subjects, indicating that prediabetic subjects had a higher rate of general obesity (based on BMI) and central obesity (based on WHR). Both systolic blood pressure and diastolic blood pressure were significantly increased in prediabetic subjects as compared to controls. The lipid parameters, that is, total cholesterol, triglyceride, LDL-C, and VLDL-C, were significantly increased in prediabetic subjects as compared to controls. Patients with prediabetes had significantly lowered HDL values compared with control subjects. Figure 1 and Figure 2 show comparison of CIMT and atherogenic indices between control and prediabetic subjects, respectively. The CIMT was found to be increased significantly in prediabetic subjects as compared to control subjects (0.69 ± 0.04 mm vs. 0.57 ± 0.03 mm, *p* < 0.001). Atherogenic indices, that is, CRR, AC, and AIP, were significantly increased in prediabetic subjects as compared to the controls (5.87 ± 0.87 vs. 4.23 ± 0.50, *p* < 0.001; 4.87 ± 0.87 vs. 3.23 ± 0.50, *p* < 0.001 and 0.29 ± 0.07 vs. 0.09 ± 0.09, *p* < 0.001, respectively). Table 2 shows the correlation of AIP, CRR, AC, and CIMT with cardiovascular risk factors in prediabetic subjects. AIP was significantly and positively correlated with all the cardiovascular risk factors, i.e., BMI, WHR, systolic blood pressure (SBP), diastolic blood pressure (DBP), fasting plasma glucose (FPG), two hour post glucose (2 h-PG), TC, TG, LDL, and VLDL, but not with HDL, which was negatively correlated in prediabetic subjects. CRR was significantly and positively correlated with BMI, WHR, SBP, DBP, FPG, 2 h-PG, TC, TG, LDL, and VLDL but not with HDL, which was negatively correlated. Also, there was a significant positive correlation of AC with BMI, WHR, SBP, DBP, FPG, 2 h-PG, TC, TG, LDL, and VLDL. Significant negative correlation was observed between AC and HDL. Similarly, CIMT was significantly and positively correlated with BMI, WHR, SBP, DBP, FPG, 2 h-PG, TC, TG, LDL, and VLDL. Significant negative correlation was observed between CIMT and HDL. In addition, AIP, CRR, AC, and CIMT were found to be significantly and positively correlated with age in prediabetic subjects, indicating that as the age advances, the risk of cardiovascular disease development increases. Table 3 shows the correlation of AIP, CRR, AC, and CIMT with cardiovascular risk factors in control subjects. Table 4 shows correlation of atherogenic indices with CIMT in prediabetic and control subjects. A significant and positive correlation was observed between CIMT and AIP, CRR, and AC in prediabetic subjects, whereas in control subjects, the correlation was not significant.

## 4. Discussion

Prediabetes is the intermediate state of abnormal glucose regulation that lies between normal blood glucose levels and type 2 diabetes mellitus and has been considered as a risk factor for diabetes mellitus and cardiovascular disease (CVD) [20]. In this cross-sectional study, we determined the atherogenic indices (CRR, AC, and AIP) and CIMT in prediabetic subjects and assessed the relationship between them.

Dyslipidemia plays a major role in the pathophysiology of atherosclerosis and is associated with increased risk for the development of CVD [21]. Low-density lipoprotein (LDL) is the primary atherogenic lipoprotein, whereas high-density lipoprotein (HDL) is the predominant antiatherosclerotic lipoprotein [22,23]. In our study, we found significant increased levels of TC, TG, LDL-C, and VLDL-C in prediabetic subjects as compared to controls except for HDL-C, which was significantly decreased. This dyslipidemic pattern in prediabetes is very much similar to the findings of previous studies [7,8,24]. Insulin resistance may lead to the abnormality of lipid and lipoprotein in the hyperglycemic state. There occurs hyperinsulinemia, enhanced hepatic gluconeogenesis, and glucose output in an insulin resistant status. In addition, insulin resistance decreases the suppression of lipolysis in adipose tissue, leading to high free acid flux and enhances the secretion of hepatic very low-density lipoprotein. This condition leads to increased TG and reduces the levels of HDL-C [25,26]. It is well known that the increase in TG levels increases the level of small dense LDL, which finally causes an increased risk for the development of cardiovascular disease [27]. This is due to small dense LDL particles having strong atherogenic characteristics, and they can cause atherosclerosis by increasing the process of lipid peroxidation and generating reactive oxygen species [15]. HDL reduces the peripheral cholesterol by transporting it to the liver. In addition to this, it also contains antioxidant enzymes such as paraoxonase [28]. Thus, the HDL-C is considered as antiatherogenic and may exert a protective effect on atherosclerotic heart disease [29]. Decreased HDL cholesterol levels are strongly associated with increased risk for cardiovascular disease and thickness of carotid intima-media [30]. In addition to the traditional lipid parameters, we have calculated atherogenic indices, that is, CRR, AC, and AIP. The CRR has been found to be significantly elevated in prediabetic subjects as compared to healthy controls, indicating that prediabetic subjects are at increased risk for the development of cardiovascular disease in future. The AC in the prediabetic subjects was significantly higher than that of control subjects. Non-HDL cholesterol is said to be an appropriate surrogate marker for total apolipoprotein B because of its high correlation with the apolipoprotein B levels. However, in routine clinical practices, standardized measurements of apolipoprotein B are not always available [31]. So, this simple ratio of non-HDL (atherogenic) and HDL (antiatherogenic) cholesterol could provide valuable information in identifying subjects who are at risk for cardiovascular disease [32]. Recently, AIP has been introduced, which is a mathematical relationship between TG and HDL-C and has been successfully used as an additional index for assessment of cardiovascular risk. It was shown to be a better predictor for myocardial infarction and atherosclerotic heart diseases [33,34]. This is because AIP is positively correlated with the fractional esterification rate of HDL (FERHDL) and is inversely correlated with LDL particle size. This ratio accurately reflects the presence of atherogenic small LDL and HDL particles and is considered as a sensitive predictor of coronary atherosclerosis and cardiovascular risk [35].

Indeed, it has been suggested that AIP values of −0.3 to 0.1 are associated with low, 0.1–0.24 with medium, and above 0.24 with high CV risk [36]. In our study, AIP values were significantly higher in prediabetic subjects compared to controls, which is in line with the studies done by Regmi et al. [37] and Thiyagarajan et al. [38], and an AIP value of 0.29 ± 0.07, as observed in our study, indicates that prediabetic subjects are at high risk for the development of cardiovascular disease in future.

Atherosclerosis, one of the driving forces of CVD, is a disease that progresses silently over several decades before symptoms eventually occur. Ultrasonography of the carotid artery has become a useful tool to noninvasively identify early stage atherosclerotic changes in the arterial wall. Intima-media thickness and detection of plaque formation can be used as early and sensitive indicators for early stage atherosclerosis [39,40]. As an easy and cheap marker, CIMT measurement has been widely used for the detection of subclinical atherosclerosis [41]. In several studies conducted with patients having prediabetes, it has been reported that the values of CIMT are higher in prediabetic subjects compared to healthy controls [42,43,44]. In our study, we also found that CIMT values of prediabetic subjects were increased significantly as compared to the values obtained in control subjects. A thickened CIMT does not immediately lead to cardiovascular events but reflects the degree of atherosclerosis elsewhere in the arterial system [45].

To the best of our knowledge, this was the first study that has investigated the relationship between atherogenic indices and CIMT in prediabetic subjects and found that the atherogenic indices were significantly and positively correlated with CIMT, which is the well-known marker of subclinical atherosclerosis. However, such correlations were not observed in control subjects. A strong positive correlation between CIMT and AIP has been shown in different studies, and it has been reported that AIP values can be used as a strong predictive marker of subclinical atherosclerosis [46,47,48,49]. Hence, in the present study, we demonstrated that atherogenic indices could be used for the assessment of the risk of subclinical atherosclerosis in prediabetic subjects.

The advantage of this study is that the subjects were selected on the basis of both fasting plasma glucose and 2-h plasma glucose (after giving 75 g of glucose). In addition, our study was carried out in a large sample size and was adequately powered since the significance of results was high. Despite advantages, our study has major limitations because of cross-sectional nature of the data which limits the inferences about causal relationships.

## 5. Conclusions

In conclusion, atherogenic indices, i.e., CRR, AC, and AIP, and CIMT were higher in prediabetic subjects compared with normal healthy individuals. Moreover, there was a strong correlation of these atherogenic indices with CIMT values. These results indicate greater propensity of prediabetes for the development of cardiovascular disease. Hence, atherogenic indices can be used in addition to routine lipid parameters for the better assessment of subclinical atherosclerosis in prediabetic subjects since these indices are inexpensive and can be calculated from routine lipid parameters. However, further studies are needed in order to get more precise results.

## Figures and Tables

**Table 1:** Sociodemographic and biochemical characteristics of the studied subjects.

Variables	Control Subjects (*n* = 200)	Prediabetic Subjects (*n* = 200)
Age (years)	39.07 ± 7.60	39.90 ± 8.84 ^NS^
Sex (M/F)	112/88	106/94 ^NS^
BMI (kg/m^2^)	23.42 ± 0.94	29.08 ± 1.66 **
WC (cm)	82.51 ± 2.82	92.02 ± 3.91 **
HC (cm)	95.42 ± 2.42	95.99 ± 2.61 *
WHR	0.86 ± 0.03	0.96 ± 0.03 **
SBP (mmHg)	116.30 ± 3.31	127.00 ± 5.66 **
DBP (mmHg)	77.48 ± 3.36	81.87 ± 3.63 **
FPG (mg/dL)	90.19 ± 3.83	116.13 ± 3.62 **
2-h PG (mg/dL)	123.65 ± 6.20	150.13 ± 4.16 **
TC (mg/dL)	180.19 ± 14.33	190.67 ± 18.44 **
TG (mg/dL)	120.70 ± 19.84	148.19 ± 18.80 **
HDL (mg/dL)	42.86 ± 3.40	32.81 ± 2.79 **
LDL (mg/dL)	113.20 ± 14.47	128.22 ± 18.60 **
VLDL (mg/dL)	24.14 ± 3.97	29.64 ± 3.76 **

BMI: Body mass index; WC: Waist circumference; HC: Hip circumference; WHR: Waist-to-hip ratio; SBP: Systolic blood pressure; DBP: Diastolic blood pressure; FPG: Fasting plasma glucose; 2-h PG: 2-h post glucose; TC: Total cholesterol; TG: Triglyceride; HDL: High-density lipoprotein; LDL: Low-density lipoprotein; VLDL: Very low-density lipoprotein; ^NS^ Not significant; * Significant at *p* < 0.05; ** Significant at *p* < 0.001.

**Table 2:** Shows correlation of AIP, CRR, AC, and CIMT with cardiovascular risk factors in prediabetic subjects.

Variables	AIP	CRR	AC	CIMT
Age	0.378 **	0.422 **	0.422 **	0.419 **
BMI	0.385 **	0.426 **	0.426 **	0.340 **
WHR	0.236 **	0.251 **	0.251 **	0.242 **
SBP	0.369 **	0.365 **	0.365 **	0.441 **
DBP	0.311 **	0.392 **	0.392 **	0.244 **
FPG	0.607 **	0.636 **	0.636 **	0.559 **
2-h PG	0.569 **	0.590 **	0.590 **	0.653 **
TC	0.340 **	0.825 **	0.825 **	0.395 **
HDL	−0.682 **	−0.755 **	−0.755 **	−0.385 **
TG	0.878 **	0.341 **	0.341 **	0.449 **
LDL	0.261 **	0.862 **	0.862 **	0.359 **
VLDL	0.878 **	0.341 **	0.341 **	0.449 **

BMI: Body mass index; WHR: Waist-to-hip ratio; SBP: Systolic blood pressure; DBP: Diastolic blood pressure; FPG: Fasting plasma glucose; 2-h PG: 2-h post glucose; TC: Total cholesterol; TG: Triglyceride; HDL: High-density lipoprotein; LDL: Low-density lipoprotein; VLDL: Very low-density lipoprotein; CRR: Cardiac risk ratio; AC: Atherogenic coefficient; AIP: Atherogenic index of plasma; CIMT: Carotid intima-media thickness; ** Significant at *p* < 0.01 (2-tailed).

**Table 3:** Shows correlation of AIP, CRR, AC, and CIMT with cardiovascular risk factors in control subjects.

Variables	AIP	CRR	AC	CIMT
Age	0.003 ^NS^	0.084 ^NS^	0.083 ^NS^	0.325 **
BMI	0.138 ^NS^	0.108 ^NS^	0.108 ^NS^	−0.004 ^NS^
WHR	0.050 ^NS^	0.011 ^NS^	0.011 ^NS^	0.006 ^NS^
SBP	0.092 ^NS^	0.052 ^NS^	0.053 ^NS^	−0.009 ^NS^
DBP	−0.015 ^NS^	0.127 ^NS^	0.127 ^NS^	0.053 ^NS^
FPG	0.092 ^NS^	0.112 ^NS^	0.112 ^NS^	0.116 ^NS^
2-h PG	0.081 ^NS^	0.051 ^NS^	0.051 ^NS^	0.049 ^NS^
TC	0.150 *	0.712 **	0.712 **	0.050 ^NS^
HDL	−0.542 **	−0.711 **	−0.711 **	−0.068 ^NS^
TG	0.917 **	0.254 **	0.255 **	0.059 ^NS^
LDL	0.024 ^NS^	0.802 **	0.802 **	0.050 ^NS^
VLDL	0.917 **	0.254 **	0.255 **	0.059 ^NS^

BMI: Body mass index; WHR: Waist-to-hip ratio; SBP: Systolic blood pressure; DBP: Diastolic blood pressure; FPG: Fasting plasma glucose; 2-h PG: 2-h post glucose; TC: Total cholesterol; TG: Triglyceride; HDL: High-density lipoprotein; LDL: Low-density lipoprotein; VLDL: Very low-density lipoprotein; CRR: Cardiac risk ratio; AC: Atherogenic coefficient; AIP: Atherogenic index of plasma; CIMT: Carotid intima-media thickness; ^NS^ Not significant; * Significant at *p* < 0.05 (2-tailed); ** Significant at *p* < 0.01 (2-tailed).

**Table 4:** Shows correlation of atherogenic indices with CIMT in prediabetic and control subjects.

Variables	CIMT	
	Prediabetic Subjects	Control Subjects
AIP	0.529 **	0.075 ^NS^
CRR	0.495 **	0.081 ^NS^
AC	0.495 **	0.081 ^NS^

CRR: Cardiac risk ratio; AC: Atherogenic coefficient; AIP: Atherogenic index of plasma; CIMT: Carotid intima-media thickness; ** Significant at *p* < 0.01 (2-tailed); ^NS^ Not significant.

## References

[1] Deedwania P.C., Fonseca V.A. (2005). Diabetes, prediabetes and cardiovascular risk: Shifting the paradigm. Am. J. Med..

[2] Ford E.S., Zhao G., Li C. (2010). Pre-diabetes and the risk for cardiovascular disease: A systematic review of the evidence. J. Am. Coll. Cardiol..

[3] Tabak A.G., Herder C., Rathmann W., Brunner E.J., Kivimaki M. (2012). Prediabetes: A high-risk state for diabetes development. Lancet.

[4] International Diabetes Federation (2015). IDF Diabetes Atlas.

[5] Anjana R.M., Deepa M., Pradeepa R., Mahanta J., Narain K., Das H.K., Adhikari P., Rao P.V., Saboo B., Kumar A. (2017). Prevalence of diabetes and prediabetes in 15 states of India: Results from the ICMR-INDIAB population-based cross-sectional study. Lancet Diabetes Endocrinol..

[6] Grundy S.M. (2012). Pre-diabetes, metabolic syndrome, and cardiovascular risk. J. Am. Coll. Cardiol..

[7] Kansal S., Kamble T.K. (2016). Lipid profile in prediabetes. J. Assoc. Physicians India.

[8] Balgi V., Harshavardan L., Sahna E., Thomas S.K. (2017). Pattern of lipid profile abnormality in subjects with prediabetes. Int. J. Sci. Study.

[9] Millan J., Pinto X., Munoz A., Zuniga M., Rubies-Prat J., Pallardo L.F., Masana L., Mangas A., Hernández-Mijares A., González-Santos P. (2009). Lipoprotein ratios: Physiological significance and clinical usefulness in cardiovascular prevention. Vasc. Health Risk Manag..

[10] Bafna A., Maheshwari R.S., Ved R.K., Sarkar P.D., Batham A.R. (2012). Study of atherogenic indices in nephrotic syndrome. Int. J. Biol. Med. Res..

[11] Deric M., Kojic-Damjanov S., Cabarkapa V., Eremic N. (2008). Biochemical Markers of Atherosclerosis. JMB.

[12] Onat A. (2004). Lipids, lipoproteins and apolipoproteins among Turks, and impact on coronary heart disease. Anadolu Kardiyol. Derg..

[13] Liu T., Meng X.Y., Li T., Zhang D.Y., Zhou Y.H., Han Q.F., Wang L.H., Wu L., Yao H.C. (2016). Rosuvastatin may stabilize vulnerable carotid plaques and reduce carotid intima media thickness in patients with hyperlipidemia. Int. J. Cardiol..

[14] Hemerich D., van der Laan S.W., Tragante V., den Ruijter H.M., de Borst G.J., Pasterkamp G., de Bakker P.I., Asselbergs F.W. (2015). Impact of carotid atherosclerosis loci on cardiovascular events. Atherosclerosis.

[15] Cure M.C., Tufekci A., Cure E., Kirbas S., Ogullar S., Kirbas A., Unal H., Yuce S., Cakmak S. (2013). Low-density lipoprotein subfraction, carotid artery intima-media thickness, nitric oxide, and tumor necrosis factor alpha are associated with newly diagnosed ischemic stroke. Ann. Indian Acad. Neurol..

[16] Mookadam F., Moustafa S.E., Lester S.J., Warsame T. (2010). Subclinical atherosclerosis: Evolving the role of carotid intima-media thickness. Prev. Cardiol..

[17] American Diabetes Association (2017). Classification and diagnosis of diabetes. Sec. 2. In Standards of Medical Care in Diabetes-2017. Diabetes Care.

[18] Mahat R.K., Singh N., Gupta A., Rathore V. (2018). Oxidative DNA damage and carotid intima media thickness as predictors of cardiovascular disease in prediabetic subjects. J. Cardiovasc. Dev. Dis..

[19] Friedewald W.T., Levy R.I., Fredrickson D.S. (1972). Estimation of the concentration of low-density lipoprotein cholesterol in plasma, without use of the preparative ultracentrifuge. Clin. Chem..

[20] Huang S., Peng W., Zhao W., Sun B., Jiang X. (2017). Higher plasma C-reactive protein and lower plasma adiponectin are associated with increased carotid artery intima-media thickness in patients with impaired glucose regulation. Asian Biomed..

[21] Yang C., Sun Z., Li Y., Ai J., Sun Q., Tian Y. (2014). The correlation between serum lipid profile with carotid intima-media thickness and plaque. BMC Cardiovasc. Disord..

[22] Nakamura H., Arakawa K., Itakura H., Kitabatake A., Goto Y., Toyota T., Nakaya N., Nishimoto S., Muranaka M., Yamamoto A. (2006). Primary prevention of cardiovascular disease with pravastatin in Japan (MEGA Study): A prospective randomised controlled trial. Lancet.

[23] Cannon C.P., Braunwald E., McCabe C.H., Rader D.J., Rouleau J.L., Belder R., Joyal S.V., Hill K.A., Pfeffer M.A., Skene A.M. (2004). Intensive versus moderate lipid lowering with statins after acute coronary syndromes. N. Engl. J. Med..

[24] Gholi Z., Heidari-Beni M., Feizi A., Iraj B., Askari G. (2016). The characteristics of pre-diabetic patients associated with body composition and cardiovascular disease risk factors in the Iranian population. J. Res. Med. Sci..

[25] Boden G., Laakso M. (2004). Lipids and glucose in type 2 diabetes: What is the cause and effect?. Diabetes Care.

[26] McGarry J.D. (2002). Banting lecture 2001: Dysregulation of fatty acid metabolism in the etiology of type 2 diabetes. Diabetes.

[27] Boizel R., Benhamou P.Y., Lardy B., Laporte F., Foulon T., Halimi S. (2000). Ratio of triglycerides to HDL cholesterol is an indicator of LDL particle size in patients with type 2 diabetes and normal HDL cholesterol levels. Diabetes Care.

[28] Gaal K., Tarr T., Lorincz H., Borbas V., Seres I., Harangi M., Fülöp P., Paragh G. (2016). High-density lipopoprotein antioxidant capacity, subpopulation distribution and paraoxonase-1 activity in patients with systemic lupus erythematosus. Lipids Health Dis..

[29] Krauss R.M. (2004). Lipids and lipoproteins in patients with type 2 diabetes. Diabetes Care.

[30] Rosvall M., Persson M., Östling G., Nilsson P.M., Melander O., Hedblad B., Engström G. (2015). Risk factors for the progression of carotid intima-media thickness over a 16-year follow-up period: The Malmö Diet and Cancer Study. Atherosclerosis.

[31] (2002). Third Report of the National Cholesterol Education Program (NCEP) Expert Panel on Detection, Evaluation and Treatment of High Blood Cholesterol in Adults (Adult Treatment Panel III) Final Report. Circulation.

[32] Singh M., Pathak M.S., Paul A. (2015). A study on atherogenic indices of pregnancy induced hypertension patients as compared to normal pregnant women. J. Clin. Diagn. Res..

[33] Onat A., Can G., Kaya H., Hergenc G. (2010). “Atherogenic index of plasma” (log10 triglyceride/high-density lipoprotein-cholesterol) predicts high blood pressure, diabetes, and vascular events. J. Clin. Lipidol..

[34] Dobiasova M. (2004). Atherogenic index of plasma [log(triglycerides/HDL-cholesterol)]: Theoretical and practical implications. Clin. Chem..

[35] Dobiasova M., Frohlich J. (2001). The plasma parameter log (TG/HDL-C) as an atherogenic index: Correlation with lipoprotein particle size and esterification rate in apoB-lipoprotein-depleted plasma (FERHDL). Clin. Biochem..

[36] Dobiasova M. (2006). AIP-atherogenic index of plasma as a significant predictor of cardiovascular risk: From research to practice. Vnitr. Lek..

[37] Regmi P., Baral B., Raut M., Khanal M.P. (2016). Atherogenic index of plasma for prediction of future cardiovascular disease in prediabetes and diabetes population. Atherosclerosis.

[38] Thiyagarajan R., Subramanian S.K., Sampath N., Trakroo M., Pal P., Bobby Z., Paneerselvam S., Das A.K. (2012). Association between cardiac autonomic function, oxidative stress and inflammatory response in impaired fasting glucose subjects: Cross-sectional study. PLoS ONE.

[39] O'Leary D.H., Polak J.F., Kronmal R.A., Manolio T.A., Burke G.L., Wolfson S.K. (1999). Carotid-artery intima and media thickness as a risk factor for myocardial infarction and stroke in older adults. Cardiovascular Health Study Collaborative Research Group. N. Engl. J. Med..

[40] Lorenz M.W., Markus H.S., Bots M.L., Rosvall M., Sitzer M. (2007). Prediction of clinical cardiovascular events with carotid intima-media thickness: A systematic review and meta-analysis. Circulation.

[41] Icli A., Cure E., Cure M.C., Uslu A.U., Balta S., Mikhailidis D.P., Ozturk C., Arslan S., Sakız D., Sahin M. (2016). Endocan levels and subclinical atherosclerosis in patients with systemic lupus erythematosus. Angiology.

[42] Altin C., Sade L.E., Gezmis E., Ozen N., Duzceker O., Bozbas H., Eroglu S., Muderrisoglu H. (2016). Assessment of subclinical atherosclerosis by carotid intima-media thickness and epicardial adipose tissue thickness in prediabetes. Angiology.

[43] Karbek B., Cakal E., Cakir E., Bozkurt N., Unsal I., Sahin M., Delibasi T. (2013). Cardiovascular risk factors, carotid artery intima media thickness, and HSCRP levels in patients with impaired glucose metabolism. Minerva Endocrinol..

[44] Aydin Y., Berker D., Ustün I., Gül K., Erden G., Kutlucan A., Yilmaz Aydin L., Güler S. (2011). Evaluation of carotid intimamedia thickness in impaired fasting glucose and impaired glucose tolerance. Minerva Endocrinol..

[45] Touboul P.J., Grobbee D.E., den Ruijter H. (2012). Assessment of subclinical atherosclerosis by carotid intima media thickness: Technical issues. Eur. J. Prev. Cardiol..

[46] Yildiz G., Duman A., Aydin H., Yilmaz A., Hür E., Mağden K., Cetin G., Candan F. (2013). Evaluation of association between atherogenic index of plasma and intima-media thickness of the carotid artery for subclinic atherosclerosis in patients on maintenance hemodialysis. Hemodial. Int..

[47] Cure E., Icli A., Ugur Uslu A., Aydoğan Baykara R., Sakiz D., Ozucan M., Yavuz F., Arslan S., Cumhur Cure M., Kucuk A. (2017). Atherogenic index of plasma may be strong predictor of subclinical atherosclerosis in patients with Behçet disease. Z. Rheumatol..

[48] Uslu A.U., Kucuk A., Icli A., Cure E., Sakiz D., Arslan S., Baykara R.A. (2017). Plasma atherogenic index is an independent indicator of subclinical atherosclerosis in systemic lupus erythematosus. Eurasian J. Med..

[49] Icli A., Cure E., Uslu A.U., Sakiz D., Cure M.C., Ozucan M., Baykara R.A., Karakoyun A., Balta S., Ozturk C. (2017). The relationship between atherogenic index and carotid artery atherosclerosis in familial mediterranean fever: A pilot study. Angiology.

